# What Changes Have Occurred in Opioid Prescriptions and the Prescribers of Opioids Before TKA and THA? A Large National Registry Study

**DOI:** 10.1097/CORR.0000000000002653

**Published:** 2023-04-26

**Authors:** Heather E. van Brug, Rob G. H. H. Nelissen, Frits R. Rosendaal, Eveline L. A. van Dorp, Marcel L. Bouvy, Albert Dahan, Maaike G. J. Gademan

**Affiliations:** 1 Department of Orthopaedics, Leiden University Medical Center, Leiden, the Netherlands; 2 Department of Clinical Epidemiology, Leiden University Medical Center, Leiden, the Netherlands; 3 Department of Anaesthesiology, Leiden University Medical Center, Leiden, the Netherlands; 4 Utrecht Institute for Pharmaceutical Sciences, Division of Pharmacoepidemiology and Clinical Pharmacology, Utrecht University, Utrecht, the Netherlands

## Abstract

**Background:**

Opioid use before TKA or THA is linked to a higher risk of revision surgery and less functional improvement. In Western countries, the frequency of preoperative opioid use has varied, and robust information on temporal changes in opioid prescriptions over time (in the months before surgery as well as annual changes) and among prescribers is necessary to pinpoint opportunities to improve on low-value care patterns, and when they are recognized, to target physician populations for intervention strategies.

**Questions/purposes:**

(1) What proportion of patients undergoing arthroplasties receive an opioid prescription in the year before TKA or THA, and what were the preoperative opioid prescription rates over time between 2013 and 2018? (2) Does the preoperative prescription rate vary between 12 and 10 months and between 3 and 1 months in the year before TKA or THA, and did it change between 2013 and 2018? (3) Which medical professionals were the main prescribers of preoperative opioids 1 year before TKA or THA?

**Methods:**

This was a large-database study drawn from longitudinally maintained national registry sources in the Netherlands. The Dutch Foundation for Pharmaceutical Statistics was linked to the Dutch Arthroplasty Register from 2013 to 2018. TKAs and THAs performed because of osteoarthritis in patients older than 18 years, which were also uniquely linked by age, gender, patient postcode, and low–molecular weight heparin use, were eligible. Between 2013 and 2018, 146,052 TKAs were performed: 96% (139,998) of the TKAs were performed for osteoarthritis in patients older than 18 years; of them, 56% (78,282) were excluded because of our linkage criteria. Some of the linked arthroplasties could not be linked to a community pharmacy, which was necessary to follow patients over time, leaving 28% (40,989) of the initial TKAs as our study population. Between 2013 and 2018, 174,116 THAs were performed: 86% (150,574) were performed for osteoarthritis in patients older than 18 years, one arthroplasty was excluded because of an outlier opioid dose, and a further 57% (85,724 of 150,574) were excluded because of our linkage criteria. Some of the linked arthroplasties could not be linked to a community pharmacy, leaving 28% (42,689 of 150,574) of THAs, which were performed between 2013 and 2018. For both TKA and THA, the mean age before surgery was 68 years, and roughly 60% of the population were women. We calculated the proportion of patients undergoing arthroplasties who had at least one opioid prescription in the year before arthroplasty and compared data from 2013 to 2018. Opioid prescription rates are given as defined daily dosages and morphine milligram equivalents (MMEs) per arthroplasty. Opioid prescriptions were assessed by preoperative quarter and by operation year. Possible changes over time in opioid exposure were investigated using linear regression, adjusted for age and gender, in which the month of operation since January 2013 was used as the determinant and MME as the outcome. This was done for all opioids combined and per opioid type. Possible changes in opioid prescription rates in the year before arthroplasty were assessed by comparing the time period of 1 to 3 months before surgery with the other quarters. Additionally, preoperative prescriptions per operation year were assessed per prescriber category: general practitioners, orthopaedic surgeons, rheumatologists, and others. All analyses were stratified by TKA or THA.

**Results:**

The proportion of patients undergoing arthroplasties who had an opioid prescription before TKA increased from 25% (1079 of 4298) in 2013 to 28% (2097 of 7460) in 2018 (difference 3% [95% CI 1.35% to 4.65%]; p < 0.001), and before THA increased from 25% (1111 to 4451) to 30% (2323 to 7625) (difference 5% [95% CI 3.8% to 7.2%]; p < 0.001). The mean preoperative opioid prescription rate increased over time between 2013 and 2018 for both TKA and THA. For TKA, an adjusted monthly increase of 3.96 MME was observed (95% CI 1.8 to 6.1 MME; p < 0.001). For THA, the monthly increase was 3.8 MME (95% CI 1.5 to 6.0; p = 0.001. For both TKA and THA, there was a monthly increase in the preoperative oxycodone rate (3.8 MME [95% CI 2.5 to 5.1]; p < 0.001 and 3.6 [95% CI 2.6 to 4.7]; p < 0.001, respectively). For TKA, but not for THA, there was a monthly decrease in tramadol prescriptions (-0.6 MME [95% CI -1.0 to -0.2]; p = 0.006). Regarding the opioids prescribed in the year before surgery, there was a mean increase of 48 MME (95% CI 39.3 to 56.7 MME; p < 0.001) for TKA between 10 and 12 months and the last 3 months before surgery. For THA, this increase was 121 MME (95% CI 110 to 131 MME; p < 0.001). Regarding possible differences between 2013 and 2018, we only found differences in the period 10 to 12 months before TKA (mean difference 61 MME [95% CI 19.2 to 103.3]; p = 0.004) and the period 7 to 9 months before TKA (mean difference 66 MME [95% CI 22.0 to 110.9]; p = 0.003). For THA, there was an increase in the MMEs prescribed between 2013 and 2018 for all four quarters, with mean differences ranging from 43.9 to 55.4 MME (p < 0.05). The average proportion of preoperative opioid prescriptions prescribed by general practitioners ranged between 82% and 86% (41,037 of 49,855 for TKA and 49,137 of 57,289 for THA), between 4% and 6% (2924 of 49,855 for TKA and 2461 of 57,289 for THA), by orthopaedic surgeons, 1% by rheumatologists (409 of 49,855 for TKA and 370 of 57,289 for THA), and between 9% and 11% by other physicians (5485 of 49,855 for TKA and 5321 of 57,289 for THA). Prescriptions by orthopaedic surgeons increased over time, from 3% to 7% for THA (difference 4% [95% CI 3.6 to 4.9]) and 4% to 10% for TKA (difference 6% [95% CI 5% to 7%]; p < 0.001).

**Conclusion:**

Between 2013 and 2018, preoperative opioid prescriptions increased in the Netherlands, mainly because of a shift to more oxycodone prescriptions. We also observed an increase in opioid prescriptions in the year before surgery. Although general practitioners were the main prescribers of preoperative oxycodone, prescriptions by orthopaedic surgeons also increased during the study period. Orthopaedic surgeons should address opioid use and its associated negative effects in preoperative consultations. More intradisciplinary collaboration seems important to limit the prescribing of preoperative opioids. Additionally, research is necessary to assess whether opioid cessation before surgery reduces the risk of adverse outcomes.

**Level of Evidence:**

Level III, therapeutic study.

## Introduction

Against international guidelines, opioids are often prescribed for patients with pain from osteoarthritis (OA). Because opioids have limited effects on chronic pain in patients with noncancer pain [1], they are considered low-value care in OA treatment [9]. Moreover, recently, opioids showed similar effects on pain relief and disability reduction to a placebo drug in a trial in patients with OA [24].

Recently, in the Netherlands, we found an increase in opioid prescriptions after TKA and THA between 2013 and 2018 [23]. The highest postoperative prescription rates were observed among patients who underwent arthroplasty after receiving a preoperative prescription. Other research likewise showed an association between preoperative opioid use and chronic opioid use [17]. It is therefore important to assess the changes in preoperative opioid prescriptions in patients undergoing TKA and THA. Previous studies have reported differences in the use of opioids before knee and hip arthroplasty, with a proportion ranging from 10% in Finland to 60% in the United States [11, 12, 18, 19, 22, 27]. However, most studies did not focus on changes over time; instead, they made comparisons between the different operation years (such as 2010 versus 2015) or focused on opioid use in multiple years before arthroplasty surgery (for example, from 5 years before surgery until the year before surgery) [11, 12, 18, 19, 22]. Furthermore, no studies that we know of have focused on possible changes in the months or weeks before arthroplasty. An assessment of opioid prescriptions before surgery may give information on how the level of preoperative pain is perceived because a patient’s anxiety about the surgical procedure could impact pain levels [25]. This is important because in that period, patients may have increased joint complaints and may visit a doctor more frequently. The preoperative period therefore could be a possible target for evaluating patients’ preoperative opioid use and to encourage opioid cessation in patients who are using them to reduce the risks related to opioid use and revision rates [14]. To address the latter, information on who prescribes opioids is important to possibly prevent the use or overuse of opioids. In the Netherlands, it is estimated that in the general population, 75% of primary prescriptions and 90% of repeat prescriptions come from general practitioners [20]. In the United States, orthopaedic surgeons prescribed 11% to 15% of preoperative opioid prescriptions, and family physicians prescribed roughly one-third [15]. However, because of differences in healthcare systems, data from European countries (such as the Netherlands) are important.

We therefore investigated: (1) What proportion of patients undergoing arthroplasties receive an opioid prescription in the year before TKA or THA, and what were the preoperative opioid prescription rates over time between 2013 and 2018? (2) Does the preoperative prescription rate vary between 12 and 10 months and between 3 and 1 months in the year before TKA or THA, and did it change between 2013 and 2018? (3) Which medical professionals were the main prescribers of preoperative opioids 1 year before TKA or THA?

## Patients and Methods

### Study Design and Setting

We performed a comparative study drawn from high-quality, longitudinally maintained, national registry sources in the Netherlands in which we linked two national databases, the Dutch Arthroplasty Register (LROI) and the Dutch Foundation for Pharmaceutical Statistics (SFK).

### Data Sources

We obtained data on THAs and TKAs and most patient demographics from the LROI. The LROI covers all hospitals performing arthroplasties in the Netherlands. The completeness of primary THA and TKA data are more than 98% [6].

We obtained pharmaceutical dispensing data from the SFK, which contains data from more than 95% of the community pharmacies, including outpatient pharmacies. Individual-level opioid dispensing data were derived 1 year before arthroplasty, including Anatomic-Therapeutic-Chemical codes, dose, and number dispensed.

### Data Linkage

Because of Dutch privacy legislation, the SFK did not have an individual identifier such as a social security number to link the databases. Thus, we used deterministic data linkage to link data from the SFK to the LROI. The LROI and SFK datasets were linked based on a combination of birth year, gender, and four-digit postcode (patient or hospital). In addition, the surgery date was linked to thromboprophylaxis medication (if it was dispensed 4 days before to 10 days after surgery) because surgery data were unavailable in the SFK. Both low–molecular weight heparin or direct oral anticoagulation prescribed close to the surgery date were used as a thromboprophylaxis link. In the Netherlands, both types of thromboprophylaxis are fully reimbursed by insurance. After linking the databases, several quality checks were performed: The data linkage was checked for implausible results (such as dispensed medication after death), we assessed the representativeness of the linked population against the source population, and we performed external validation to ensure linkage quality. Data quality was highest among arthroplasties linked by low–molecular weight heparin and patient postcode (data not shown). This group also showed a good resemblance to the population from which the arthroplasties originated (Supplemental Table 1; http://links.lww.com/CORR/B82). We observed some minor differences: our linked population was somewhat younger and consisted of less women.

The external validation was performed with data from Netherlands Statistics. We compared the number of patients undergoing arthroplasties with at least one opioid prescription in an operation year in our linked database (LROI-SFK) with results found in Netherlands Statistics. These proportions were quite similar and showed a similar increase over time. These results were described in the supplementary material of our published study on postoperative prescription rates [23].

### Patients

Patients older than 18 who underwent arthroplasties because of OA who were uniquely linked by patient postcode and who were prescribed low–molecular weight heparin were deemed eligible. We found that patients linked by direct oral anticoagulation and hospital postcode were more likely to have implausible links when checked. Arthroplasties were also excluded if they were only linked to an outpatient pharmacy because these could not be followed over time. Hence, in these cases, it was not possible to trace them to the patients’ local community pharmacies. Arthroplasties with more than 4000 defined daily doses in the year before surgery were also excluded.

Between 2013 and 2018, 146,052 TKAs were performed. A total of 96% (139,998) of these arthroplasties were performed because of OA in patients older than 18 years; of those, 41% (57,274 of 139,998) were excluded because they were not uniquely linked or not linked by patient postcode. Another 25% (21,008 of 82,724) were excluded because they were not linked to low–molecular weight heparin use; 34% (20,727 of 61,716) of these arthroplasties were excluded because they were linked to outpatient pharmacies, leaving 29% (40,989 of 139,998) of the TKAs performed because of OA in patients older than 18 years.

**Fig. 1:**
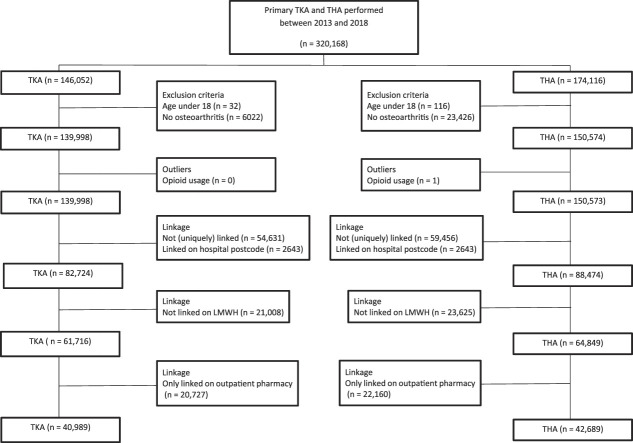
This flowchart shows the patient selection for the study. LMWH = low–molecular weight heparin.

Between 2013 and 2018, 174,116 THAs were performed. We included 86% (150,574 of 174,116) of these arthroplasties because of OA in patients older than 18 years; one arthroplasty was excluded because of an outlier opioid dose. Of the remaining, 41% (62,099 of 150,573) were excluded because they were not uniquely linked or not linked by patient postcode. Another 27% (23,625 of 88,474) were excluded because they were not linked to low–molecular weight heparin use. Additionally, 34% (22,160 of 64,849) of these arthroplasties were excluded because they were linked to outpatient pharmacies, leaving 28% (42,689 of 150,574) of the arthroplasties performed because of OA in patients older than 18 years (Fig. 1).

### Participants’ Baseline Characteristics

The mean age for TKA was 68 ± 9 years, and it was 68 ± 10 years for THAs. Sixty percent (24,566 of 40,989) of all TKAs and 61% (26,146 of 42,689) of THAs were performed in women. Seventy-nine percent (32,524 of 40,989) of TKAs and 65% (27,709 of 42,689) of THAs were performed in patients with a BMI greater than 25 kg/m^2^ (Table 1).

**Table 1.** Population characteristics stratified by primary TKAs and THAs

Characteristic		TKA (n = 40,989)		THA (n = 42,689)	
Age in years		68 ± 9		68 ± 10	
Women		60 (24,566)		61 (26,146)	
BMI in kg/m^2a^					
Underweight (≤ 18.5)		0.1 (53)		1 (238)	
Normal weight (18.5 to 25)		15 (6222)		29 (12,352)	
Overweight (25 to 30)		39 (15,947)		41 (17,606)	
Obesity (30 to 40)		37 (15,171)		23 (9647)	
Morbid obesity (> 40)		3 (1406)		1 (456)	
Missing		5 (2190)		6 (2390)	
ASA class					
I		14 (5857)		19 (8052)	
II		69 (28,095)		66 (28,057)	
III-IV		17 (6940)		15 (6493)	
Missing		0.2 (97)		0.2 (87)	
Socioeconomic status					
Low		14 (5598)		11 (4814)	
Below average		19 (7969)		18 (7808)	
Average		38 (15,438)		38 (16,192)	
Above average		24 (9899)		26 (11,299)	
High		5 (1851)		6 (2374)	
Missing		0.6 (234)		0.5 (202)	
Patients who smoked^a^		9 (3559)		10 (4482)	
Missing		10 (4188)		10 (4350)	
Charnley classification^a,b^					
A		37 (15,226)		40 (17,225)	
B1		31 (12,695)		27 (11,429)	
B2		17 (7156)		19 (8197)	
C		2 (883)		2 (795)	
Not applicable		0.3 (107)		0.2 (105)	
Missing		12 (4922)		12 (4938)	
Operation year					
2013		4298		4451	
2014		7318		7533	
2015		7587		7825	
2016		7051		7460	
2017		7275		7795	
2018		7460		7625	
Prosthesis fixation method					
Cemented		92 (37,797)		22 (9422)	
Uncemented		5 (2014)		69 (29,425)	
Hybrid		3 (1120)		9 (3795)	
Missing		0.1 (58)		0.1 (47)	

Data presented as mean ± SD or % (n).

a Available since 2014.

b Charnley classification: A = one joint affected with osteoarthrosis, B1 = two joints affected (both hips or both knees), B2 = contralateral joint with a prothesis, C = multiple joints affected with osteoarthrosis or a chronic disease impairing quality of life (in walking).

### Descriptive Data

The following patient data were included: age in years, gender, BMI, current smoking status (yes or no), OA as the indication for surgery (yes or no), American Society of Anesthesiologists (ASA) classification, and the Charnley score. The ASA classification was used to categorize a patient’s physical fitness before surgery (I [healthy] to IV [severe systemic disease, constant threat to life]). The Charnley score categorizes the OA degree in other joints (A [one joint affected] to C [multiple joints affected or chronic disease that limits quality of life]). Socioeconomic status was based on individual four-digit postcodes from the SFK. Socioeconomic status originated from the 2014 and 2016 measurements of the Netherlands Institute for Social Research, based on income, education, and occupation of Dutch inhabitants, and was received from the SFK. Socioeconomic status scores were based on quintiles: low (≤ -1.5), below average (-1.49 to -0.5), average (-0.49 to 0.49), above average (0.5 to 1.49), and high (≥ 1.5).

The following prosthesis-related information was derived: joint (knee or hip) and fixation (cemented, uncemented, or hybrid).

### Opioid Prescriptions

Opioids were classified following the Anatomic-Therapeutic-Chemical-5 classification [26]. Opioid use before arthroplasty was defined as at least one dispensed opioid prescription from a Dutch pharmacy in the year before arthroplasty. Opioid exposure was expressed as defined daily doses and morphine milligram equivalents (MMEs). Defined daily doses were defined as the supplied dose divided by the average maintenance dose according to the WHO Collaborating Centre for Drug Statistics Methodology. MMEs were determined by calculating the dosages of each opioid prescription and multiplying this by an MME conversion factor. For example, 1 mg of morphine equals 1 MME, 1 mg of oxycodone equals 1.5 MME, and 1 mg of tramadol equals 0.1 MME. If no MME or defined daily doses existed for a certain opioid (such as codeine with acetaminophen), it was not counted. Furthermore, opioid prescriptions before arthroplasty were categorized per year and week based on the time of prescribing.

Opioid prescribers were divided into the following categories: general practitioners, orthopaedic surgeons, rheumatologists, and others.

### Ethical Approval

Ethical approval was waived by the Medical Ethics Committee of Leiden-Den Haag-Delft (reference number: G19.018) because this research was not subject to the Dutch Medical Research Act (Wet medisch-wetenschappelijk onderzoek met mensen [WMO]) as we used existing register data.

### Statistical Analysis

All analyses were stratified by TKA and THA. The population characteristics are described using descriptive statistics. Continuous outcomes are shown as mean with SD and categorical outcomes are shown as a proportion per category. Characteristics of the LROI population and the linked arthroplasties were evaluated to assess representativeness. Descriptive statistics were also used to assess yearly opioid prescriptions before arthroplasty for each year from 2013 to 2018; the proportion of patients with at least one opioid prescription before surgery was calculated and compared using chi-square tests. Opioid exposure in the total population and for the five most frequently prescribed opioids was expressed as MME/arthroplasty; means and 95% confidence intervals (CIs) around the point estimate are given. We performed linear regression analyses with the month of operation since January 2013 as the determinant and MME as the outcome to assess whether there was an increase in prescribed MMEs over time. We did this for all opioid types together as well as separately for each opioid. The analyses were adjusted for gender and age.

For each week (within the year) before surgery, the MMEs per week were calculated for the entire population and for the opioid user group. This was done by adding all prescriptions and dividing it by the number of arthroplasties performed in that year. We fitted a locally estimated scatterplot smoothing curve (estimation function to create a smoothed curve with 95% CIs) to visualize the weekly change in opioid dose per arthroplasty. Additionally, for each preoperative quarter (within the year before surgery) we calculated the change in MME from the quarter 12 to 10 months before surgery to the quarter 3 months before surgery using paired t-tests, because of the dependent nature of the prescriptions. The change in the operation years was assessed by comparing all four quarters between 2013 and 2018 by using independent t-tests.

For each preoperative quarter (within the year before surgery), we calculated the number of patients undergoing arthroplasties who had a prescription to assess whether the opioid dose or the number of arthroplasties with an opioid prescription was responsible for an increase in opioid exposure. The change in the number of operations per quarter was assessed using the McNemar test for paired proportions. Furthermore, for each prescriber category, we calculated the proportion of opioid prescriptions per operation year.

All data cleaning and analyses were performed in R 3.6.2 (R Foundation for Statistical Computing).

### Sensitivity Analysis

The analyses of preoperative opioid prescriptions were also performed in a subset of index arthroplasties (the first performed arthroplasty recorded in our data) to assess the possible bias we introduced by using all arthroplasties (including ones performed at the same time or within 1 year of another).

## Results

### Preoperative Opioid Prescriptions and Use Changes in the Year Before TKA or THA

**Fig. 2:**
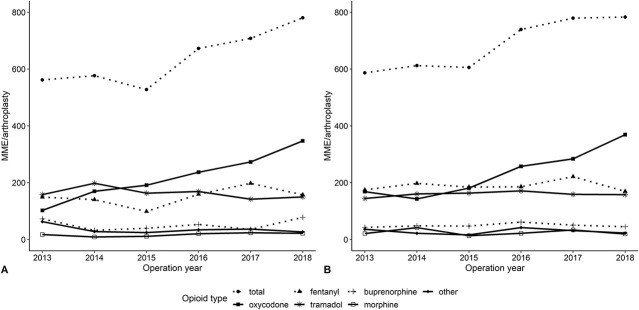
These graphs show opioid prescriptions over time in the year before (**A**) TKA and (**B**) THA in MME.

The proportion of TKAs with at least one opioid prescription in the year before surgery fluctuated from 25% (1079 of 4298) in 2013 to 28% (2097 of 7460) in 2018 (difference 3% [95% CI 1.35% to 4.65%]; p < 0.001) (Supplemental Table 2; http://links.lww.com/CORR/B83). The total opioid exposure increased from 562 MME/arthroplasty (95% CI 422 to 702) in 2013 to 781 MME/arthroplasty (95% CI 664 to 896) in 2018 (Fig. 2A). Per month, an increase of 4.0 MMEs was observed (95% CI 1.8 to 6.1; p < 0.001) (Table 2). For individual opioids, oxycodone prescriptions increased from 102 MME/arthroplasty (95% CI 51 to 154) to 347 MME/arthroplasty (95% CI 270 to 424) between 2013 and 2018, with a monthly increase of 3.8 MME (95% CI 2.4 to 5.1; p < 0.001). In 2013, oxycodone comprised 16% (760 of 4683) of opioids prescribed before TKA; this increased to 46% (4660 of 10,043) in 2018 (difference 30% [95% CI 29% to 32%]; p < 0.001). Tramadol prescriptions decreased from 158 MME/arthroplasty (95% CI 134 to 182) to 150 MME/arthroplasty (95% CI 133 to 167), with a monthly decrease of -0.6 MME (95% CI -1.0 to -0.2; p = 0.01).

**Table 2.** The association between the month of surgery and the total MMEs in the preoperative year before TKA and THA

	TKA		THA	
	Adjusted change per month^a^	p value	Adjusted change per month^a^	p value
Overall	4.0 (1.8 to 6.1)	< 0.001	3.8 (1.5 to 6.0)	0.001
Oxycodone	3.8 (2.5 to 5.1)	< 0.001	3.6 (2.6 to 4.7)	< 0.001
Tramadol	-0.6 (-1.0 to -0.2)	0.006	0.0 (-0.4 to 0.4)	0.96
Morphine	0.2 (-0.0 to 0.5)	0.10	-0.1 (-0.6 to 0.4)	0.66
Fentanyl	0.6 (-0.7 to 1.9)	0.37	0.2 (-1.4 to 1.8)	0.80
Buprenorphine	0.2 (-0.5 to 1.0)	0.54	0.0 (-0.4 to 0.5)	0.86
Other^b^	-0.3 (-0.6 to 0.1)	0.13	-0.0 (-0.3 to 0.3)	0.88

All values reported as beta (95% CI).

a Adjusted for age and gender.

b Codeine with paracetamol, hydromorphone, tapentadol, pentazocine, pethidine.

Among opioid users, the mean MMEs prescribed in the preoperative year was 2239 MMEs (95% CI 1693 to 2784) in 2013 and 2777 MMEs in 2018 (95% CI 2377 to 3177). Before THA, the prevalence of opioid prescription in the year before surgery increased from 25% (1111 of 4451) in 2013 to 30% (2323 of 7625) in 2018 (difference 5% [95% CI 3.8% to 7.2%]; p < 0.001) (Supplemental Table 2; http://links.lww.com/CORR/B83). The total opioid exposure increased from 587 MME/arthroplasty (95% CI 464 to 710) to 783 MME/arthroplasty (95% CI 683 to 883) between 2013 and 2018 (Fig. 2B). Per month, there was an increase of 3.8 MME (95% CI 1.5 to 6.0; p = 0.001). For individual opioids, oxycodone prescriptions increased from 168 MME/arthroplasty (95% CI 99 to 238) in 2013 to 369 MME/arthroplasty (95% CI 301 to 436) in 2018, with a monthly increase of 3.6 MMEs (95% CI 2.6 to 4.7; p < 0.001) (Table 2). In 2013, oxycodone comprised 20% (1006 of 5034) of opioid prescriptions, and in 2018, this increased to 46% (5439 of 11,770) (difference 26% [95% CI 25% to 28%]; p < 0.001). Exposure to all other opioids remained relatively similar. Among opioid users, the opioid exposure before THA was 2351 MME/arthroplasty (95% CI 1872 to 2830) in 2013 and 2571 MME/arthroplasty (95% CI 2254 to 2887) in 2018. The results for defined daily doses showed similar increases (Supplemental Digital Content 1; http://links.lww.com/CORR/B84).

### Weekly Preoperative Prescription Rates in the Year Before TKA or THA

**Fig. 3:**
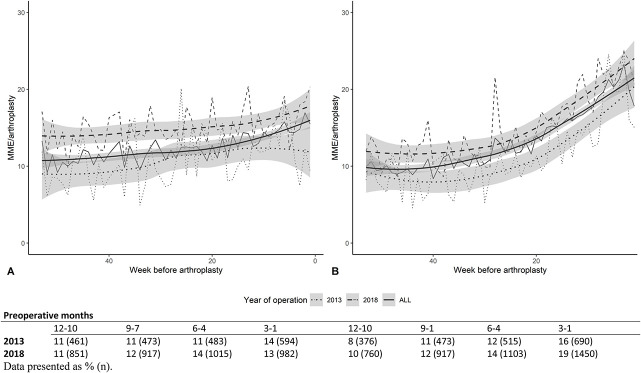
These graphs show preoperative prescribed MMEs per week in patients who underwent (**A**) TKA and (**B**) THA.

We depicted the prescribed MMEs over time with a smoothed curve in 2013 and 2018 as well as for all years together (Fig. 3), with the numbers of users for each quarter depicted underneath.

The mean increase for TKA between the 12- to 10-month period before surgery and the 3- to 1-month period closest to surgery was 48.0 MME (95% CI 39.3 to 56.7; p < 0.001) (Supplemental Table 3; http://links.lww.com/CORR/B85). Between 2013 and 2018, there was a difference in the 12- to 10-month period 1 year before TKA (mean difference 61.2 MME [95% CI 19.2 to 103.3]; p = 0.004), whereas no difference was observed in the 3- to 1-month period before surgery (mean difference: 52.6 MME [95% CI -0.3 to 105.5]; p = 0.05) (Supplemental Table 4; http://links.lww.com/CORR/B86).

The mean increase for THA between the 12- to 10-month period before surgery and the 3- to 1-month period was 120.5 MME (95% CI 110.1 to 131.0; p < 0.001) (Supplemental Table 3; http://links.lww.com/CORR/B85).

Between 2013 and 2018, there was an increase in the MMEs prescribed between 2013 and 2018 in the 12- to 10-month period (mean difference 50.7 MME [95% CI 8.7 to 92.6]; p = 0.02) and the 3- to 1-month period (mean difference 55.4 MME [95% CI 0.9 to 109.9]; p = 0.046) (Supplemental Table 4; http://links.lww.com/CORR/B86). Defined daily doses showed similar increases (Supplemental Digital Content 1; http://links.lww.com/CORR/B84).

### Prescribers of Preoperative Opioids Within 1 Year of TKA or THA

**Fig. 4:**
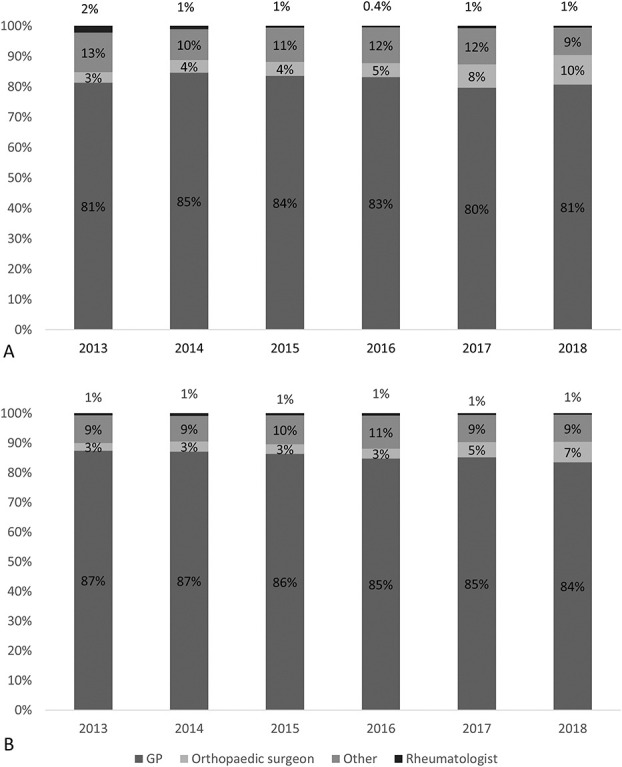
These graphs show the proportion of preoperative opioid prescribers before (**A**) TKA and (**B**) THA per operation year; GP = general practitioner.

The proportion of preoperative opioid prescriptions prescribed by general practitioners was 82% (41,037 of 49,855) for TKA and 86% (49,137 of 57,289) for THA, and it was 6% (2924 of 49,855) for TKA and 4% (2461 of 57,289) for THA by orthopaedic surgeons; it was 1% by rheumatologists (409 of 49,855 for TKA and 370 of 57,289 for THA), and it was 11% (5485 of 49,855) for TKA and 9% (5321 of 57,289) for THA by other physicians. Prescriptions by orthopaedic surgeons increased over time for TKA (4% [164 of 4683] to 10% [956 of 10,043], difference 6% [95% CI 5% to 7%]; p < 0.001) (Fig. 4A) and THA (3% to 7%, difference 4% [95% CI 3.6 to 4.9]). In the 3 months before surgery, except for 2013, orthopaedic surgeons prescribed fewer prescriptions than in the fourth, third, and second quarters (12 to 3 months before TKA) (Supplemental Table 5; http://links.lww.com/CORR/B87). The proportion of prescriptions from orthopaedic surgeons before THA seemed stable over the different quarters (Supplemental Table 6; http://links.lww.com/CORR/B88).

### Sensitivity Analyses

A total of 78% (31,800 of 40,989) of the TKAs and 80% (34,012 of 42,689) of the THAs of our study population were index arthroplasties. In these index arthroplasties, the proportion of TKAs with an opioid prescription seemed stable at approximately 23%, whereas the proportion of index THAs increased (24% to 28%) over the study period (Supplemental Table 2; http://links.lww.com/CORR/B83). For the preoperative prescription rates (Supplemental Fig. 1; http://links.lww.com/CORR/B89) and monthly changes from 2013 to 2018 (Supplemental Table 7; http://links.lww.com/CORR/B90), as well as the changes in opioid prescriptions in the weeks (Supplemental Fig. 2; http://links.lww.com/CORR/B91) and quarters (Supplemental Table 8; http://links.lww.com/CORR/B92) in the preoperative year, we found similar patterns as in the entire study population. The mean difference between 2013 and 2018 in the 12 to 10 months before index TKA was less than in the overall population. The same was true for the 3- to 1-month period before the index TKA and THA (Supplemental Table 9; http://links.lww.com/CORR/B93).

## Discussion

Opioid prescriptions before arthroplasty are known to increase the risk of reoperation. They have considerable side effects such as nausea and obstipation, and they may lead to addiction. Although others have recently found that postoperative opioid prescriptions increased after TKA or THA in the past 15 years [16, 21, 23], relatively little is known about time changes in preoperative opioid use in this population. In addition, knowing who prescribes opioids is important so these healthcare professionals can be targeted for strategies focusing on treatment other than opioids for pain reduction. We found an increase in the proportion of patients undergoing TKAs or THAs with a preoperative opioid prescription between 2013 and 2018. Furthermore, the overall opioid prescription rate increased for both TKA and THA. Additionally, for TKA and THA, the opioid prescription rate increased from 12 to 10 months before surgery to the last 3 months before surgery. Most opioids were prescribed by a general practitioner, although the proportion of orthopaedic surgeons as prescribers of preoperative opioids increased between 2013 and 2018.

### Limitations

This study has several limitations. First, we used dispensing data, but a dispensed drug may not have been consumed. Nonetheless, a prescribed opioid, even when not consumed, could still be a marker of low-value care in this population. Additionally, the indication for the opioid prescription was not available, and the opioids might have been prescribed for complaints or conditions other than hip or knee OA. However, our study aimed to describe the opioid prescriptions in the OA population before arthroplasty surgery without focusing on causality. Third, the registration of healthcare providers who prescribed medication, particularly hospital specialists, might have improved over time. As such, the increased proportion of orthopaedic surgeons prescribing opioids might have (partly) been an effect of this improved registration. Fourth, there were some limitations concerning our data linkage. The data linkage was based on arthroplasties and not on individuals, so some individuals could have been included multiple times. Therefore, we conducted a sensitivity analysis using only index arthroplasties, which showed a difference in the proportion of TKAs in patients with a preoperative prescription. This suggests that patients who received another prosthesis within the first year influenced the proportional increase we found in TKAs. In this sensitivity analysis, the difference we found between the preoperative quarters between 2013 and 2018 in our main analysis was no longer present, but this could have been a function of lower numbers and insufficient statistical power for that endpoint. All other sensitivity analyses provided consistent results with the initial findings. Furthermore, as the datasets lacked a unique identifier to link them directly, the resulting linkage has its uncertainty. However, we carefully performed several checks, including internal and external validation, to ascertain that the quality of linkage was sufficient.

### Preoperative Opioid Prescriptions and Use Changes in the Year Before TKA or THA

Between 2013 and 2018, the proportion of arthroplasties in patients with an opioid prescription before surgery increased from 25% to 28% for TKA and from 25% to 30% for THA. Stratified by opioid type, opioid exposure to oxycodone increased; exposure to the other opioids remained stable. These changes could have unintended but important effects. Preoperative opioid prescriptions have been associated with an increase in postoperative prescriptions [5] and reoperation after surgery [2, 3, 5]. Because oxycodone is a more potent opioid, it could also lead to more side effects. The preoperative increases were in line with the postoperative increase we found in the same arthroplasty population in the Netherlands [23], but they were less apparent. The preoperative increase in oxycodone prescriptions could be related to the reintroduction of oxycodone in postoperative guidelines [10]. Prescribers, both orthopaedic surgeons and general practitioners, might have become more accustomed to prescribing opioids to patients after arthroplasty, and they might more easily prescribe them to patients indicated for arthroplasty. However, this explanation must be considered speculative. Another cause could be the possible hesitation to prescribe NSAIDs because of adverse events commonly associated with their use [4]. NSAIDs are part of the recommendation in stepped care for OA, in conjunction with acetaminophen, lifestyle education, and exercise therapy, as well as the general concept that reasonable nonsurgical alternatives should be exhausted before considering arthroplasty surgery [8]. If general practitioners are reluctant to prescribe NSAIDs, this might lead to prescription of opioids. Therefore, a study about prescribing preferences is necessary to gain insight into the driving force behind the prescribing of opioids.

The proportion of patients undergoing arthroplasty who received a preoperative opioid prescription in this study was less than in some other countries, including Sweden, Denmark, the United States, and Canada (more than 40%) [13, 19, 22], whereas in Finland, the proportion with a preoperative opioid prescription was approximately 10% [18]. In addition to national differences in the proportion of patients undergoing arthroplasty who receive a preoperative opioid prescription, there are differences in the types of opioids being prescribed. It is hard to compare the MMEs prescribed in this population to those of prior studies because we provided a rate over all the operated arthroplasties, irrespective of opioid use, and most other studies assessed the proportion of prescriptions. Recent studies showed that in England, Finland, and Denmark, weak opioids such as tramadol, and in Sweden, strong opioids such as oxycodone, are more commonly prescribed [13, 18, 27]. These international observations over time underline the importance of analyzing national data, thus enabling a full understanding of the prescription behavior of local caregivers and, consequently, allowing the development of national policies and guidelines to curb the opioid epidemic.

Continued opioid use but not intermittent use seems to be associated with an increased risk of surgical site infection, dislocation, and revision arthroplasty [19]. Hence, mitigation strategies should not only focus on the consumption of opioids, but also should consider the dosing strategy. Overall, caregivers should avoid opioids in patients before arthroplasty as much as possible, and, if opioids are prescribed before arthroplasty and these are used chronically, caregivers should try to help patients to discontinue their use before arthroplasty, when possible.

### Weekly Preoperative Prescription Rates in the Year Before TKA or THA

Between the 12- to 10-month period before TKA or THA to the period 3 to 1 months before, there was an increase in prescriptions over each of the quarters of that year. This increase in opioid prescriptions, just before the surgical date, may be a function of disease progression but could also be caused by a possible increase of anxiety in patients before a large surgical procedure (such as the replacement of a total joint) and its perceived risks, including infection, or a fear of anesthesia [7], which in turn could influence the patients’ pain perception [25]. Further, patients might have increased contact with specialists (orthopaedic surgeon or general practitioner) in the months before surgery, who might have tried to treat patients’ pain while they were waiting to be operated, despite international guidelines advising against this [1]. This increase in opioid prescriptions before surgery supports observations from other areas in Europe (such as the United Kingdom, Sweden, and Finland) [18, 27].

### Prescribers of Preoperative Opioids Within 1 Year of TKA or THA

Lastly, general practitioners most often prescribed preoperative opioids. However, there has recently been a slight increase in the number of prescriptions by orthopaedic surgeons. Based on this, orthopaedic surgeons and general practitioners should be urged to limit their preoperative prescribing of opioids. In addition to trying to limit the opioid prescriptions orthopaedic surgeons write, they also should be aware that general practitioners might also be prescribing opioids to their patients preoperatively. Therefore, orthopaedic surgeons should discuss opioid use during preoperative and postoperative consultations. Research from other countries showed similar results, namely that most preoperative prescriptions came from general practitioners and fewer came from orthopaedic surgeons [15]. This suggests the general practitioner should be the main instigator of opioid cessation strategies.

### Conclusion

In the Netherlands, between 2013 and 2018, the preoperative opioid prescription rates for patients undergoing arthroplasty increased, mainly caused by an increase in oxycodone prescriptions. In 2013 and 2018, opioid doses increased in the quarters leading up to the actual surgical date. Further monitoring of opioid prescriptions is warranted as a modifiable preoperative risk factor that impacts patients’ postoperative risks. A better understanding of why patients are being prescribed opioids in the preoperative year and what incentivizes their prescribers is necessary and must be explored further.

## Supplementary Material

SUPPLEMENTARY MATERIAL
