# Supplementary material for: What Changes Have Occurred in Opioid Prescriptions and the Prescribers of Opioids Before TKA and THA? A Large National Registry Study
Source: Clin Orthop Relat Res. 2023 Apr 26;481(9):1716–28. doi: 10.1097/CORR.0000000000002653 (PMC10427048; doi:10.1097/CORR.0000000000002653)
Supplement: SUPPLEMENTARY MATERIAL [file abjs-481-1716-s002.docx]

**Supplemental Digital Content 1**. Results expressed in defined daily doses

*Preoperative Opioid Prescription Changes in the Year Before TKA or THA*

The total opioid exposure increased before TKA from 8.2 (95% CI 6.8 to 9.5) defined daily doses per arthroplasty in 2013 to 10.4 (95% CI 9.2 to 11.5) defined daily doses per arthroplasty in 2018 (Appendix Fig. 1A). Per month, an increase of 0.03 defined daily doses was observed (95% CI 0.0 to 0.05; p = 0.04). For individual opioids, we observed a monthly increase in oxycodone prescriptions (0.03 defined daily doses [95% CI 0.02 to 0.05]; p < 0.001) and a monthly decrease in tramadol prescriptions (-0.02 defined daily doses [95% CI -0.03 to -0.01]; p = 0.01) (Appendix Table 1). Oxycodone prescriptions increased from 0.9 (95% CI 0.4 to 1.4) defined daily doses per arthroplasty to 3.1 (95% CI 2.4 to 3.8) defined daily doses, whereas tramadol prescriptions decreased from 5.3 (95% CI 4.5 to 6.1) to 5.0 (95% CI 4.4 to 5.6) defined daily doses between 2013 and 2018. Among opioid users, the opioid exposure in defined daily doses before TKA was 39.2 (95% CI to 33.6 to 44.8) defined daily doses per arthroplasty in 2013 and 38.5 (95% CI 34.6 to 42.3) in 2018. The total opioid exposure, expressed as mean defined daily doses per arthroplasty, increased before THA from 8.4 (95% CI 7.2 to 9.1) defined daily doses per arthroplasty to 10.7 (95% CI 9.7 to 11.7) defined daily doses per arthroplasty between 2013 and 2018 (Appendix Fig. 1B). Per month, an increase of 0.04 defined daily doses was observed (95% CI 0.01 to 0.05; p = 0.003). For individual opioids, we observed a monthly increase in oxycodone prescriptions (0.03 [95% CI 0.02 to 0.04]; p < 0.001) (Appendix Table 1). Oxycodone prescriptions increased from 1.5 (95% CI 0.9 to 2.1) defined daily doses per arthroplasty in 2013 to 3.3 (95% CI 2.7 to 3.9) defined daily doses per arthroplasty in 2018. Exposure to all other opioids remained relatively similar. Among opioid users, the opioid exposure in defined daily doses before THA was 40.1 (95% CI 35.0 to 45.1) defined daily doses per arthroplasty in 2013 and 36.1 (95% CI 32.9 to 39.3) in 2018.

*Weekly Preoperative Prescription Rates in the Year Before TKA or THA*

We depicted the prescribed defined daily doses over time with a smoothed curve in the years 2013 and 2018 as well as for all years together (Appendix Fig. 2). The average increase for TKA between the 12 to 10 months before surgery and the 3- to 1-month period closest to surgery was 0.74 defined daily doses (95% CI 0.65 to 0.84; p < 0.001) (Appendix Table 2). Between 2013 and 2018, there was a difference in the 12- to 10-month period 1 year before TKA (mean difference 0.76 defined daily doses [95% CI 0.33 to 1.19]; p < 0.001), whereas no difference was observed for the 3- to 1-month period (mean difference 0.50 defined daily doses [95% CI -0.02 to 1.01]; p = 0.06) (Appendix Table 3).

The average increase for THA between the 12- to 10-month period before surgery and the 3- to 1-month period was 1.8 defined daily doses (95% CI 1.7 to 1.9]; p < 0.001) (Appendix Table 2). Between 2013 and 2018, there was an increase in the MMEs prescribed between 2013 and 2018 in the 12- to 10-month period (mean difference 0.6 defined daily doses [95% CI 0.1 to 1.0]; p = 0.01) and 3- to 1-month period (mean difference: 0.6 defined daily doses [95% CI 0.03 to 1.13 defined daily doses]; p = 0.04) (Appendix Table 3).


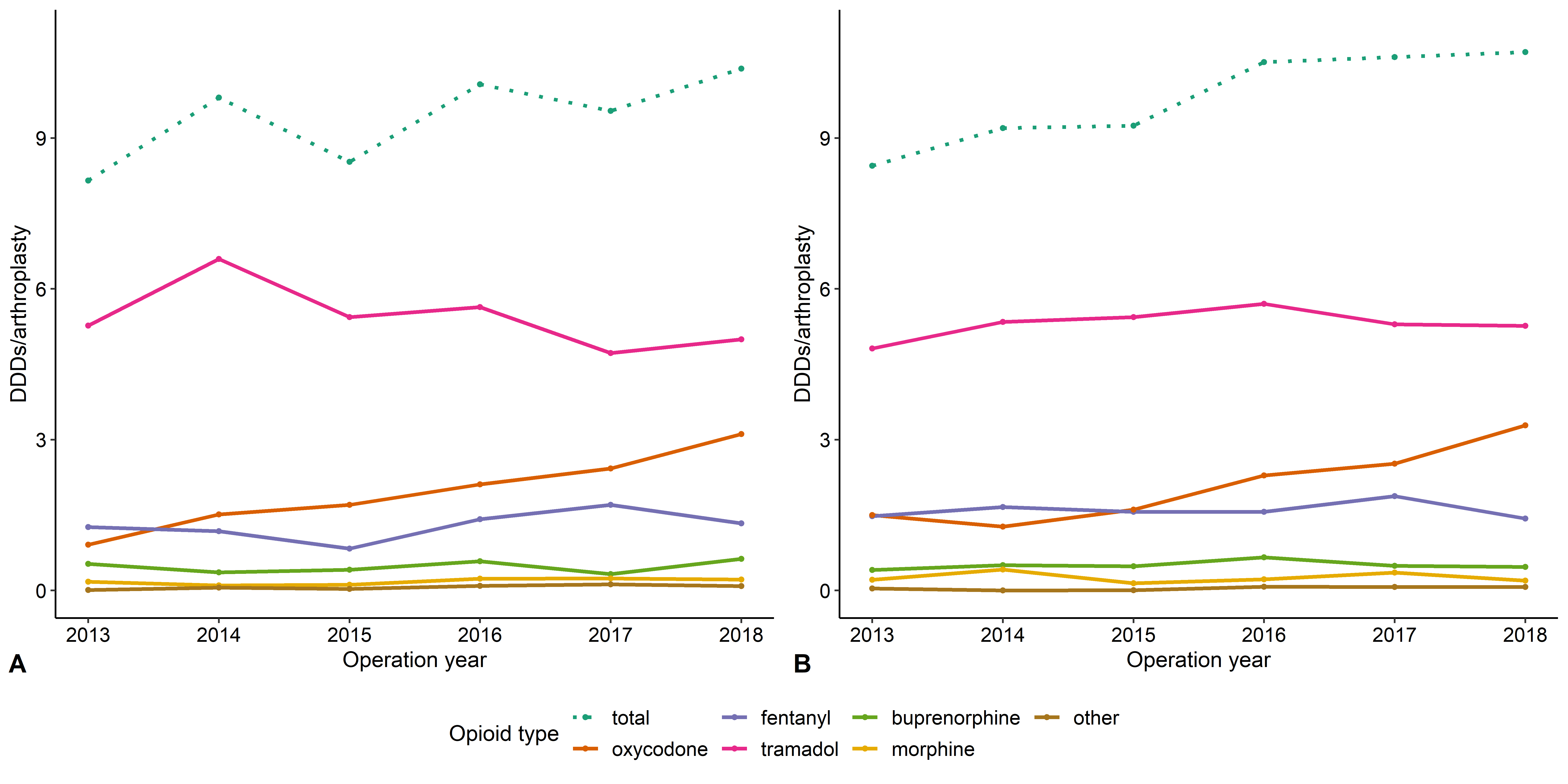


**Appendix Fig. 1** (A) This graph shows opioid prescriptions over time in the year before TKA in defined daily doses. (B) This graph shows opioid prescriptions over time in the year before THA in defined daily doses; DDDs = defined daily doses.

**Appendix Table 1.** The association between the month of surgery and the total defined daily dose in the preoperative year before total knee and hip arthroplasty

| **TKA** | | | | | **THA** | | | |
| --- | --- | --- | --- | --- | --- | --- | --- | --- |
|  | **Change per month (95% CI)** | **p value** | **Adjusted change per month (95% CI)^a^** | **p value** | **Change per month (95% CI)** | **p value** | **Adjusted change per month (95% CI)^a^** | **p value** |
| Overall | 0.02 (0.00 to 0.05) | 0.04 | 0.03 (0.00 to 0.05) | 0.04 | 0.04 (0.01 to 0.06) | 0.003 | 0.04 (0.01 to 0.05) | 0.003 |
| Oxycodone | 0.04 (0.02 to 0.05) | < 0.001 | 0.03 (0.02 to 0.05) | < 0.001 | 0.03 (0.02 to 0.04) | < 0.001 | 0.03 (0.02 to 0.04) | < 0.001 |
| Tramadol | -0.02 (-0.04 to -0.01) | 0.004 | -0.02 (-0.03 to -0.01) | 0.01 | 0.00 (-0.01 to 0.01) | 0.87 | 0.00 (-0.01 to 0.01) | 0.96 |
| Morphine | 0.00 (-0.00 to 0.01 | 0.11 | 0.00 (-0.00 to 0.01) | 0.11 | -0.00 (-0.01 to 0.00) | 0.99 | -0.00 (-0.01-0.00) | 0.79 |
| Fentanyl | 0.00 (-0.01 to 0.02) | 0.40 | 0.01 (-0.01 to 0.02) | 0.34 | 0.00 (-0.01 to 0.01) | 0.84 | 0.00 (-0.01 to 0.02) | 0.65 |
| Buprenorphine | 0.00 (-0.00 to 0.01) | 0.59 | 0.00 (-0.00 to 0.01) | 0.53 | 0.00 (-0.00 to 0.00) | 0.72 | 0.00 (-0.00 to 0.01) | 0.70 |
| Other^b^ | 0.00 (-0.00 to 0.00) | 0.16 | 0.00 (-0.00 to 0.00) | 0.15 | 0.00 (-0.00 to 0.00) | 0.09 | 0.00 (-0.00 to 0.00) | 0.06 |

^a^Adjusted for age and gender.

^b^Codeine met paracetamol, hydromorphone, tapentadol, pentazocine, pethidine.


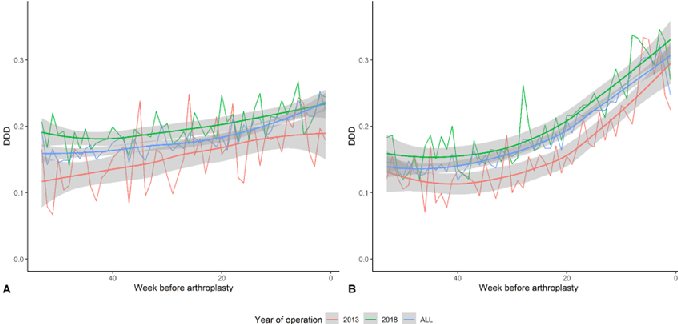


**Appendix Fig. 2** (A) This graph shows the preoperative prescribed defined daily doses per preoperative week per patients who underwent TKA. (B) This graph shows the preoperative prescribed defined daily doses per preoperative week per patients who underwent THA; DDDs = defined daily doses.

**Appendix Table 2**. The difference between the mean defined daily dose prescribed in different quarters before TKA and THA overall and stratified for 2013 and 2018

|  | **TKA** | | **THA** | |
| --- | --- | --- | --- | --- |
| **Overall** | | | | |
|  | Mean difference (95% CI) | p value | Mean difference (95% CI) | p value |
| Q4 vs Q3 | 0.15 (0.08 to 0.22) | < 0.001 | 0.21 (0.14 to 0.27) | < 0.001 |
| Q4 vs Q2 | 0.20 (0.12 to 0.28) | < 0.001 | 0.54 (0.47 to 0.61) | < 0.001 |
| Q4 vs Q1 | 0.74 (0.65 to 0.84) | < 0.001 | 1.77 (1.65 to 1.88) | < 0.001 |
| **2013** | | | | |
| Q4 vs Q3 | 0.24 (0.04 to 0.44) | 0.21 | 0.09 (-0.08 to 0.26) | 0.32 |
| Q4 vs Q2 | 0.43 (0.18 to 0.68) | < 0.001 | 0.50 (0.31 to 0.69) | < 0.001 |
| Q4 vs Q1 | 0.81 (0.53 to 1.08) | < 0.001 | 1.69 (1.31 to 2.06) | < 0.001 |
| **2018** | | | | |
| Q4 vs Q3 | 0.09 (-0.06 to 0.23) | 0.23 | 0.05 (-0.10 to 0.21) | 0.50 |
| Q4 vs Q2 | 0.18 (0.02 to 0.34) | 0.03 | 0.57 (0.3800.75) | < 0.001 |
| Q4 vs Q1 | 0.55 (0.32 to 0.77) | < 0.001 | 1.72 (1.46 to 1.98) | < 0.001 |

**Appendix Table 3**. The mean difference between defined daily dose in different preoperative quarters before TKA and THA between 2013 and 2018

|  | **TKA** | | **THA** | |
| --- | --- | --- | --- | --- |
|  | **Mean difference (95% CI)** | **p value** | **Mean difference (95% CI)** | **p value** |
| 12-10 months | 0.76 (0.33 to 1.19) | < 0.001 | 0.56 (0.12 to 1.0) | 0.01 |
| 9-7 months | 0.61 (0.13 to 0.91) | 0.007 | 0.52 (0.13 to 0.91) | 0.009 |
| 6-4 months | 0.36 (-0.17 to 0.88) | 0.18 | 0.59 (0.15 to 1.04) | 0.009 |
| 3-1 months | 0.50 (-0.02 to 1.01) | 0.06 | 0.59 (0.03 to 1.13) | 0.04 |
