# Supplementary material for: What Changes Have Occurred in Opioid Prescriptions and the Prescribers of Opioids Before TKA and THA? A Large National Registry Study
Source: Clin Orthop Relat Res. 2023 Apr 26;481(9):1716–28. doi: 10.1097/CORR.0000000000002653 (PMC10427048; doi:10.1097/CORR.0000000000002653)
Supplement: SUPPLEMENTARY MATERIAL [file abjs-481-1716-s003.docx]

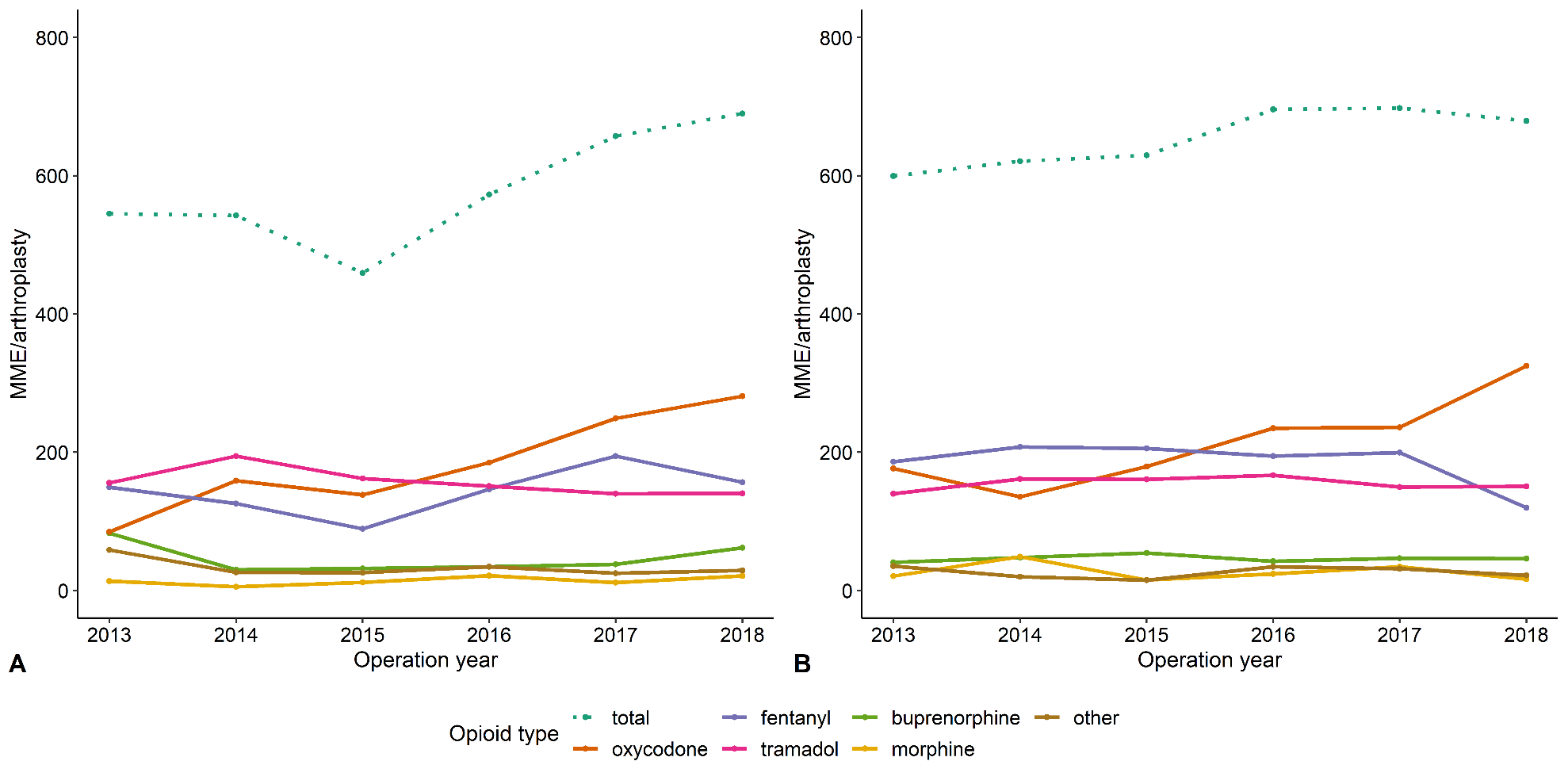


**Supplementary Fig. 1** (A) This graph shows opioid prescriptions overtime in the year before index TKA in MMEs. (B) This graph shows opioid prescriptions overtime in the year before index THA in MMEs.
