# Supplementary material for: What Changes Have Occurred in Opioid Prescriptions and the Prescribers of Opioids Before TKA and THA? A Large National Registry Study
Source: Clin Orthop Relat Res. 2023 Apr 26;481(9):1716–28. doi: 10.1097/CORR.0000000000002653 (PMC10427048; doi:10.1097/CORR.0000000000002653)
Supplement: SUPPLEMENTARY MATERIAL [file abjs-481-1716-s004.docx]

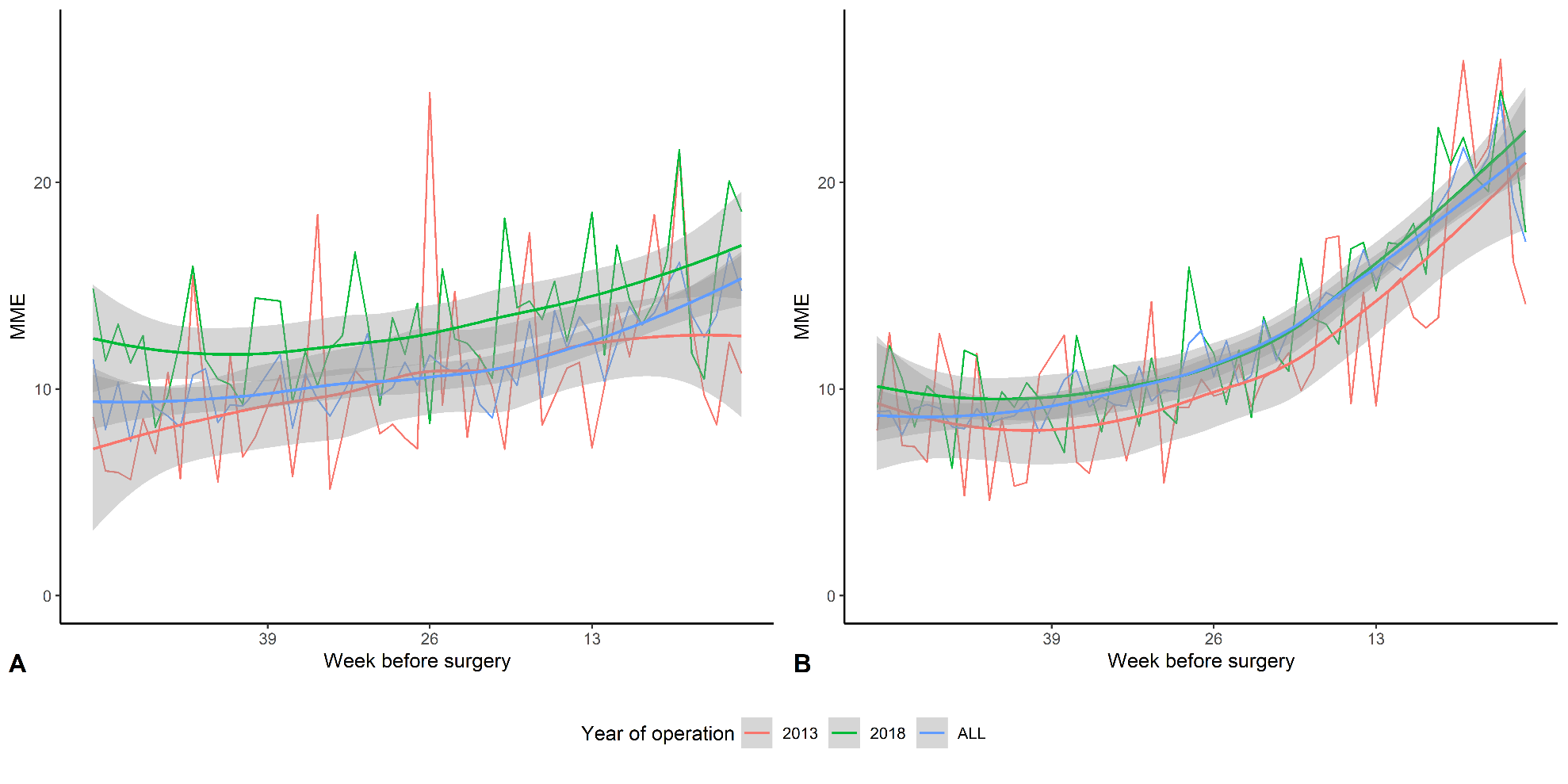
 **Supplementary Fig. 2** (A) The preoperative prescribed MMEs per preoperative week per patients who underwent index TKA is shown here. (B) The preoperative prescribed MMEs per preoperative week per patients who underwent index THA is shown here.
